# Frequent Alteration of the Tumor Suppressor Gene *APC* in Sporadic Canine Colorectal Tumors

**DOI:** 10.1371/journal.pone.0050813

**Published:** 2012-12-10

**Authors:** Lydia Youmans, Cynthia Taylor, Edwin Shin, Adrienne Harrell, Angela E. Ellis, Bernard Séguin, Xinglai Ji, Shaying Zhao

**Affiliations:** 1 Department of Biochemistry and Molecular Biology, Institute of Bioinformatics, University of Georgia, Athens, Georgia, United States of America; 2 College of Veterinary Medicine, University of Georgia, Athens, Georgia, United States of America; 3 College of Veterinary Medicine, Oregon State University, Corvallis, Oregon, United States of America; 4 Laboratory for Conservation and Utilization of Bio-Resources & Key Laboratory for Microbial Resources of the Ministry of Education, Yunnan University, Kunming, People's Republic of China; Ohio State University Medical Center, United States of America

## Abstract

Sporadic canine colorectal cancers (CRCs) should make excellent models for studying the corresponding human cancers. To molecularly characterize canine CRC, we investigated exonic sequence mutations of *adenomatous polyposis coli* (*APC*), the best known tumor suppressor gene of human CRC, in 23 sporadic canine colorectal tumors, including 8 adenomas and 15 adenocarcinomas, via exon-resequencing analysis. As a comparison, we also performed the same sequencing analysis on 10 other genes, either located at human 5q22 (the same locus as *APC*) or 18q21 (also frequently altered in human CRC), or known to play a role in human carcinogenesis. We noted that *APC* was the most significantly mutated gene in both canine adenomas and adenocarcinomas among the 11 genes examined. Significantly, we detected large deletions of ≥10 bases, many clustered near the mutation cluster region, as well as single or two base deletions in ∼70% canine tumors of both subtypes. These observations indicate that like in the human, *APC* is also frequently altered in sporadic colorectal tumors in the dog and its alteration is an early event in canine colorectal tumorigenesis. Our study provides further evidence demonstrating the molecular similarity in pathogenesis between sporadic human and canine CRCs. This work, along with our previous copy number abnormality study, supports that sporadic canine CRCs are valid models of human CRCs at the molecular level.

## Introduction

Sporadic canine cancers should make excellent models for studying the corresponding human cancers for a number of reasons. First, companion animals such as the dog share the same environment as the human, and hence are exposed to the same carcinogens. Indeed, risk factors for cancer development in dogs include air pollutants and other environmental toxins, diet and obesity, advancing age, and other similar factors [1]. Second, these cancers are naturally occurring and heterogeneous, and hence capture the essence of sporadic human cancers, unlike most genetically modified or xenograft rodent cancer models [2]. In fact, numerous anatomic and clinical similarities have been reported for the same type of cancers between the human and the dog [3]–[9]. Additionally, the dog genome has been sequenced to 7.6-fold coverage and a relatively accurate version of its sequence assemblies is available [1], which is unlike another companion animal, the cat, whose genome has only been sequenced to 2.8-fold coverage [10], [11]. This makes many experimental and bioinformatics analyses possible with the dog, but impossible with the cat. Importantly, the dog genome is rearranged when compared to the human genome [1], [12].

Because of these advantages, many researchers, including the Canine Comparative Oncology and Genomics Consortium (www.ccogc.net) and us (i.e., we are trying to develop a dog-human comparison strategy for cancer driver-passenger distinction [12]–[15]), have been actively promoting the immense value of sporadic canine cancers in basic and clinical research [e.g.], [ see references 3]–[9], [15]. However, the contribution of sporadic canine cancers towards understanding and treating human cancers clearly hinges upon the degree of molecular homology between canine cancers and their human counterparts. Unfortunately, despite of numerous anatomic and clinical similarities being reported [3]–[9], in contrast to their human counterparts, pathogenesis mechanisms of sporadic canine cancers remain poorly understood at the molecular level. Hence, molecular characterization of sporadic canine cancers becomes essential and urgent. Towards this goal, we conducted the study reported below.

Human colorectal cancer (CRC) is one of the best-understood systems for studying the molecular mechanisms of cancer initiation and progression [16]–[33]. The tumorigenesis model proposed by Vogelstein and colleagues [17] includes alteration of individual genes such as *APC*, as well as development of genomic instability in the form of either chromosome instability (CIN) or microsatellite instability (MSI) [16]–[21], [24]–[27]. We have recently demonstrated that CIN and MSI occur in sporadic canine colorectal tumors in the same fashion as their human counterparts [15]. Regarding the *APC* gene, a previous study reported a marked decrease of the *APC* protein expression in canine malignant colorectal tumors via immunohistochemical staining [34] (another related study also reported the altered cellular location of the *β-catenin* protein in canine colorectal tumors [35], a likely result of a defective *APC*). Other than these, to the best of our knowledge, we have not identified a single publication that investigated genomic sequence mutation of *APC* in sporadic canine colorectal tumors. This drastically differs from the human, where numerous sequencing studies have been published [e.g.], [ see references 16]–[19], [ 29]–[32]. In fact, databases documenting *APC* mutations have been established (e.g., www.umd.be/APC/) [32].

To better understand *APC* abnormalities in dogs, we investigated its exonic sequence mutations in 23 sporadic canine colorectal tumors (8 adenomas and 15 adenocarcinomas) via exon-resequencing. For comparison purposes, we also performed the same sequencing analysis with 10 other genes, either located at human 5q22 (the same locus as *APC*) or 18q21 (also frequently altered in human CRC [16]–[33]), or known to play a role in human carcinogenesis (see the Results section). The study revealed that *APC*, the best-known tumor suppressor of human CRC, was the most recurrently deleted gene in both canine adenomas and adenocarcinomas. The study provides further evidence demonstrating the molecular similarity in colorectal tumorigenesis between humans and dogs. This, along with our previous CIN/MIN study [15], supports that sporadic canine CRCs are valid models of human CRCs at the molecular level.

## Methods

### Canine colorectal tissue samples

Over 23 formalin-fixed, paraffin-embedded (FFPE) canine adenomas and adenocarcinomas were provided by the William R. Pritchard Veterinary Medical Teaching Hospital of the University of California-Davis School of Veterinary Medicine (UCDSVM). In addition, fresh-frozen samples of canine colorectal tumors and normal tissues, acquired during surgery, were obtained from the same hospital of the UCDSVM, the Veterinary Teaching Hospital of the University of Georgia Athens College of Veterinary Medicine (UGACVM), as well as the Animal Cancer Tissue Repository at the Colorado State University (CSU). After washing in phosphate-buffered saline, the samples were snap-frozen in liquid nitrogen for 10 minutes and then stored at −80°C until further analyses.

Dog tumor and normal samples obtained from the CSU were collected with owner informed consent and the CSU's Institutional Animal Care and Use Committee (IACUC) approval # 2963. Only tumors samples (no normal samples) were obtained from the UGACVM and the UCDSVM. These tumor samples were from dogs that had the naturally-occurring disease, and were acquired after tumor excision or biopsy that was necessary for treatment of the animals. No treatments were altered for the purpose of this study. Being that tumor samples were acquired after tumor excision or biopsy for the purpose of treating the dogs, approval from the IACUC of the respective university (UGA or UCD) was not required.

### DNA extraction

Fresh-frozen sample cryosectioning, H&E staining, and cryomicrodissection were performed as described previously [15] to enrich tumor cells for the tumor samples and normal colon epithelial cells for the normal samples. Then, genomic DNA was extracted from the dissected tissues using the DNeasy Blood & Tissue Kit (cat. no. 69506) from QIAGEN. Genomic DNA extraction from FFPE samples was performed using the QIAGEN QIAamp DNA FFPE Tissue Kit (cat. no. 56404), following the manufacturer's instruction.

### Bi-directional exon-resequencing and mutation detection

Primer design and PCR amplification of exons of the chosen genes, as well as bi-directional sequencing of the PCR products were performed at The J. Craig Venter Institute in Maryland as previously described [23]. Base-calling using the software Phred (www.phrap.org/phredphrapconsed.html), sequence trimming with the software Lucy (www.tigr.org/software/sequencing.shtml), exon sequence assembly, as well as comparison with the published dog reference genome [1] for mutation detection were performed as described [23]. A cutoff Phred quality score of 20 was used to reduce false positives due to sequencing errors, ensuring that only high quality bases with an error rate of ≤1% were eligible for mutation findings.

## Results

### Exon-resequencing of *APC* and other genes in sporadic canine colorectal tumors

**Figure 1:**
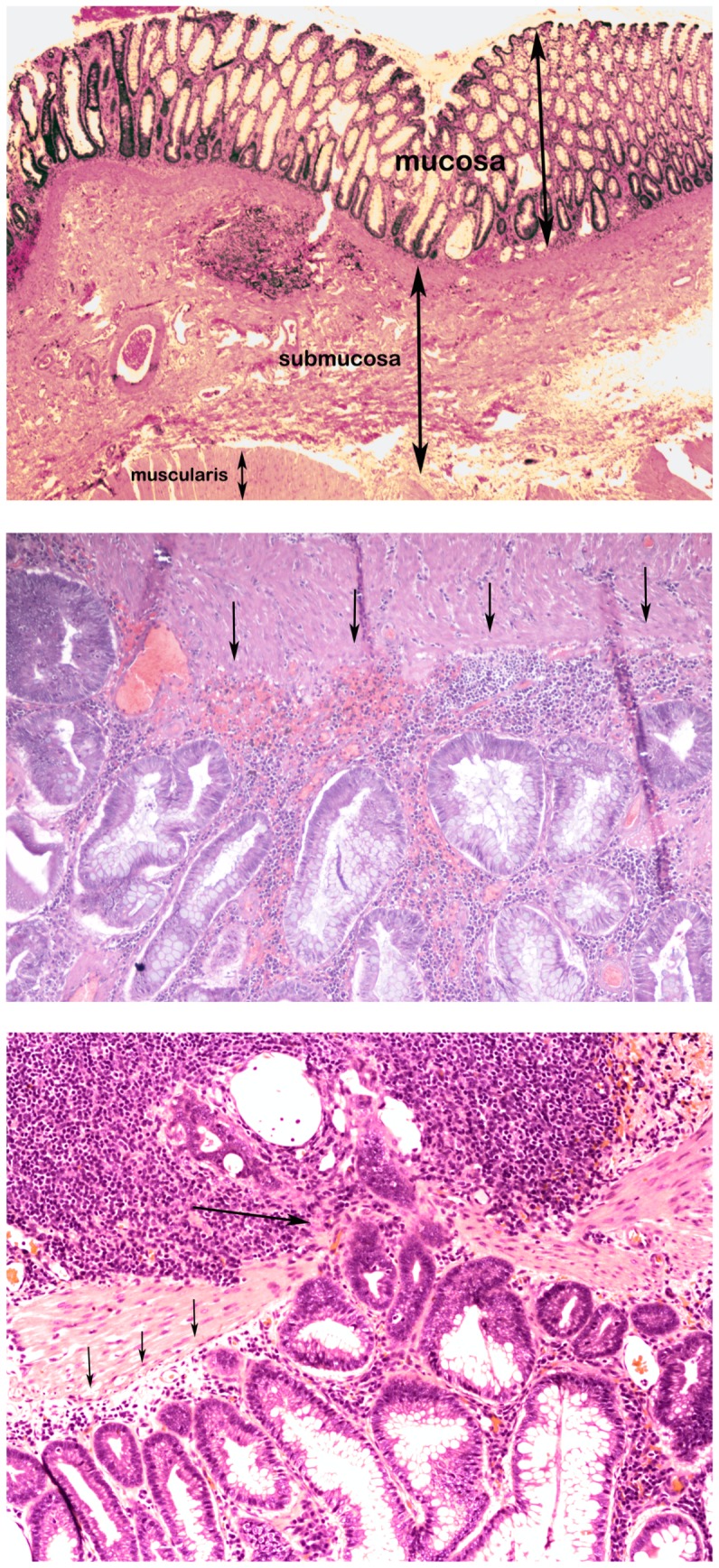
Major histopathological subtypes of canine colorectal tumors investigated. Shown from top to bottom are H&E staining images for normal colon (mucosa, submucosa, and muscle layers as indicated), colorectal adenoma, and colorectal adenocarcinoma. In the adenoma (middle), there is preservation of a distinct basement membrane (arrows). In the adenocarcinoma (bottom), a section of the basement membrane (small arrows) has been disrupted and penetrated through by neoplastic cells invading the submucosa (large arrow).

In humans, colorectal tumorigenesis is proposed to be initiated by inactivation of the *APC* gene, as its alteration is the earliest event yet identified in sporadic colorectal tumorigenesis and >85% of sporadic human colorectal tumors carry somatic mutations of *APC* [16]–[19], [29]–[32]. To investigate if these are also true in dogs, we investigated *APC* in 23 sporadic canine colorectal tumors, including 8 adenomas and 15 adenocarcinomas (Table S1), of FFPE tissue samples archived at the William R. Pritchard Veterinary Medical Teaching Hospital of the UCDSVM. As illustrated by Fig. 1, in adenomas, the basement membrane is preserved and epithelial cells proliferate only in mucosa; in invasive adenocarcinomas, however, the basement membrane is disrupted and proliferating epithelial cells spread into submucosa. Hence, similar tumor initiation and progression histological changes were observed in canine tumors as in their human counterparts.

We performed bi-directional exon-resequencing as previously described [23] to investigate sequence mutations in *APC* exons in these sporadic canine colorectal tumors. Specifically, we sequenced 14 of the 15 coding exons (as we could not design efficient primers to amplify exon 4) with 8,454 bases total for the canine *APC* gene, and were able to assemble the entire exon sequences from the forward and reverse sequences of the mostly overlapping PCR products in many cases, as described previously [23]. Combined all 23 tumors, we generated 772,729 base sequences, yielding coding exon assemblies of 140,631 bases (44,715 bases for adenomas and 95,916 bases for adenocarcinomas) in total for the canine *APC* gene (Table 1).

**Table 1:** Exonic sequence mutations of *APC* and 10 other genes in canine colorectal tumors.#

	Exons sequenced	Adenomas						
		Tumor #	Bases assembed	Bases mutated*	Bases in large deletions	Bases in indels	Bases in nonsense	Bases in missense
APC	14	8	44,715	2,429	2,134	109	11	216
10 other genes	119	8	93,000	1,266	229	127	22	612
APC/others ratio				3.99	19.38	1.79	1.04	0.73

	Exons sequenced	Adenocarcinomas						
		Tumor #	Bases assembed	Bases mutated*	Bases in large deletions	Bases in indels	Bases in nonsense	Bases in missense
**APC**	14	15	95,917	5,771	5,024	185	13	492
**10 other genes**	119	15	176,163	2,566	377	235	59	1,130
**APC/others ratio**				4.13	24.48	1.45	0.40	0.80

# See Files S1 & S2 for detailed information for each gene.

* Bases mutated include those in synonymous mutations and in UTR exons, besides those shown in here.

For comparison purposes, we also performed the same sequencing analysis on 119 exons totaling to 17,709 bases from 10 other canine genes. Seven such genes were chosen because they are located at human 5q22 (*DP1* and *MCC*), the same locus as *APC*, or 18q21 (*SMAD4, SMAD2, SMAD7, MBD1* and *MBD2*) also recurrently altered in human CRC [16]–[33]. The remaining three genes, located outside these two regions, were included because of their roles in human carcinogenesis reported in literature. These include *BIN1* (a *MYC*-interacting tumor suppressor gene [36]) and *ERCC3* (functioning in DNA excision repair [37]) at human 2q14.3, as well as *CTNNA1* (*α-catenin*, also involved in intestinal tumorigenesis [38]) at human 5q31.2. Combining all tumors together, we produced 2.7 Mb sequences, yielding coding exon assemblies of 269,163 bases (93,000 bases for adenomas and 176,163 bases for adenocarcinomas) in total for the 10 canine genes (Table 1).

We then aligned the assembled sequences to the corresponding exon reference sequence from the published dog genome [1] for mutation finding. To reduce false results that could be introduced by sequencing problems, bases with Phred quality scores of below 20 were excluded to ensure that only high quality bases with an error rate of ≤1% were eligible for mutation findings. We also exclude those changes already reported by the single nucleotide polymorphism (SNP) database (www.ncbi.nlm.nih.gov/projects/SNP/), which are mostly variations among normal dog individuals [1], [39]. Below we will describe sequence aberrations found in the canine *APC* exons and their comparison to those of the other 10 canine genes described above.

### Large deletions frequent in canine *APC* exons and clustered near the human mutation cluster region (MCR) and at the C-terminal end

Because the UCDSVM did not archive the matching normal tissue samples for these tumors, we could not perform the same sequencing analysis with the matched normal DNA. Hence, it was difficult to distinguish sequence variations existing among normal individual dogs (i.e., germline mutations), which are mostly SNPs [1], [39], from cancer-related somatic mutations. This is especially so considering that only about 3.3 million SNPs are currently released for the dog, compared to over 187 million for the human (www.ncbi.nlm.nih.gov/projects/SNP/). Consequently, we first focused on large scale changes, which often disrupt a segment of the protein and should be less frequent in coding regions among normal dogs, even for those from different breeds [40], [41] (and thus have a higher probability to be cancer-related somatic changes), compared with base substitutions [1], [39] (which could cause no changes in protein sequences - synonymous mutations, change one amino acid to another – missense mutations, or less frequently result in a premature stop codon - nonsense mutations).

**Figure 2:**
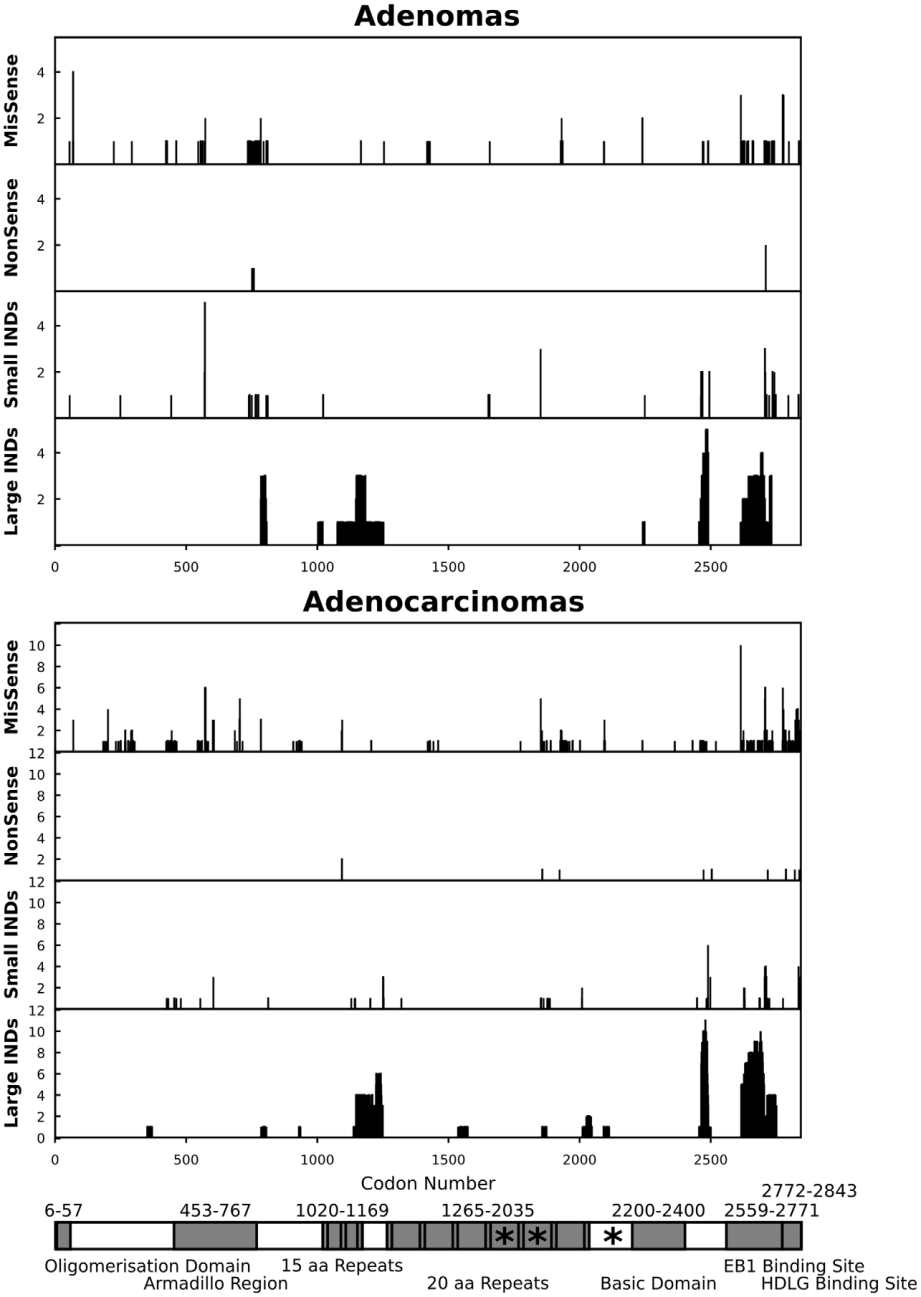
Mutation distribution along canine *APC* codons in adenomas (top) and adenocarcinomas (middle). The x-axis indicates the codon number of *APC*, and the y-axis indicates the total number of tumors altered at a specific codon for each category of missense mutations (MisSense), nonsense mutations (nonSense), small indels, and large deletions. The data indicate that large deletions were frequent and clustered in several regions of *APC* in both adenomas and adenocarcinomas (see the main text). **Bottom**: the image [modified based on reference 42] shows domains (shared area) of *APC*, corresponding to the codon number indicated above, with the stars representing SAMP repeats of the axin binding sites. The *APC* domains shown include the oligomerisation domain (codons 6–57), the armadillo region (codons 453–767), the 15 amino acid (aa) repeats (codons 1020–1169), the 20 aa repeats (codons 1265–2035), the basic domain (codons 2200–2400), the EBI binding site (codons 2559–2771), and the human disc large (HDLG) binding site (codons 2772–2843).

Consistent with our canine array comparative genome hybridization studies [15], large deletions of ≥10-bases long were frequent in canine *APC* exons, disrupting a total of 2,134 bases in 5 (63%) adenomas and 5,024 bases in 11 (73%) adenocarcinomas (Table 1). As a result, as many as 2,434 codons, 736 for adenomas and 1,698 for adenocarcinomas, were disrupted (Table 2). Except for one adenocarcinoma that carried a 57 base-deletion between the oligomerisation domain and the armadillo region, all other deletions were found downstream of the armadillo region (Fig. 2). Interestingly, these large deletions clustered in several regions of *APC* (Fig. 2). The first one, detected in the 10 tumors (3 adenomas and 7 adenocarcinomas), was within codons 1143–1250, spanning the 15amino acid (aa) repeats region and downstream (Fig. 2). It is also near the beginning of mutation cluster region (MCR), spanning codons 1286–1513, of the human *APC* gene [30], [31]. The other two clusters were found near the C-terminal end after the basic domain, one within codons 2464–2490 (found in 5 adenomas and 11 adenocarcinomas) and another within the EBI binding domain between codons 2616 to 2734 (found in 4 adenomas and 10 adenocarcinomas) (Fig. 2).

**Table 2:** Mutated codons of *APC* and other 10 genes in canine colorectal tumors.*

	Adenomas					
	Total codons assembled	Codons mutated	Codons in large deletion	Codons with indel	Codons with nonsense	Codons with missense
**APC**	14,905	951	736	60	5	133
**10 other genes**	31,000	734	83	84	17	445
**APC/others ratio**		2.69	18.44	1.49	0.61	0.62

	Adenocarcinomas					
	Total codons assembled	Codons mutated	Codons in large deletion	Codons with indels	Codons with nonsense	Codons with missense
APC	31,972	2,185	1,698	95	10	338
10 other genes	58,721	1,371	124	171	51	834
APC/others ratio		2.93	25.15	1.02	0.36	0.74

* See Files S3 & S4 for detailed information for each gene.

#### Large deletions were much less frequent in the other 10 genes sequenced

Unlike *APC* exons described above, large deletions were significantly less frequent in the other 10 genes. Specifically, only 229 bases in total were deleted for adenomas, disrupting 83 codons for 3 genes in 5 adenomas. For adenocarcinomas, 377 bases total were deleted, disrupting 124 codons for 4 genes in 11 adenocarcinomas (Tables 1 and 2). Hence, the large deletion rate is >18 times lower for adenomas and 25 times lower for adenocarcinomas for these genes, compared to *APC* (Tables 1 and 2).

Because the same samples were sequenced for all the genes, the rare occurrence of large deletions in the 10 non-*APC* genes, as described above, indicate the unlikelihood that the frequent large deletions in *APC* could be artifacts arisen from sequencing FFPE samples. Furthermore, by studying the corresponding sequences from the published dog genome [1], we observed neither repetitive sequences nor abnormal GC contents (44%, 51%, and 48%, within the normal range of exonic sequences) for the three most frequently deleted regions in *APC* shown in Fig. 2. Thus, based on their sequence contents, these regions are not prone to sequencing failure. Lastly, we performed real time quantitative PCR (qPCR) to amplify these regions with 15 fresh-frozen colorectal tumor or normal tissue samples listed in Table S2. The analysis confirmed that these regions were indeed significantly deleted in the tumors (p<0.05). In summary, these studies indicate that the frequently detected large deletions in *APC* exons are unlikely artifacts of our sequencing analysis, but are most likely cancer-related somatic changes instead.

### Small deletions frequent in canine *APC* exons and with a substantial portion found in the armadillo region

We also investigated small indels, which often result in frame-shift mutations disrupting protein function. We observed that 155 codons of diverse exons, including 60 codons from 8 adenomas (100%) and 95 codons from 14 adenocarcinomas (93%), were disrupted by small indels (i.e., single base or two base insertions/deletions) (Tables 1 and 2). In addition, clustering within the armadillo domain (codons 453–767) and around the 15 aa repeats region (codons 1020–1169), nearly one third of these indels were found at the N-terminal portion of the *APC* protein, disrupting 22 codons in 7 adenomas and 25 codons in 10 adenocarcinomas (Fig. 2). In comparison, a total of 255 codons (84 from 8 genes in 8 adenomas and 171 from 9 genes in 15 adenocarcinomas) were disrupted by small indels for the 10 non-*APC* genes combined, making the indel rate of *APC* in adenomas slightly higher (Tables 1 and 2). A bigger difference lies in the insertion/deletion ratio for these indels: while an approximately equal number of codons was disrupted by deletions and by insertions in both adenomas (47 versus 37) and adenocarcinomas (82 versus 89) for the other non-*APC* genes, significantly more codons were disrupted by deletions than by insertions for *APC*, 42 versus 18 for adenomas and 76 versus 19 for adenocarcinomas. This is consistent with the frequently observed large deletions in *APC* exons described above.

### Base substitutions in canine *APC* exons

When compared to the published canine genomic sequences [1], a total of 800 base substitutions (249 for adenomas and 551 for adenocarcinomas) were identified for *APC* exons, none of which are among the published normal dog SNPs. In comparison, 2,127 such changes (740 for adenomas and 1,387 for adenocarcinomas) were found for the 10 non-*APC* genes, with a substation rate approximately the same as *APC*. In both *APC* and non-*APC* genes, the base substitution rate is twice higher in adenocarcinomas than in adenomas. However, as shown in Table 3, the G:C→A:T transversion rate in *APC* is lower than that of the other genes (especially for adenocarcinomas), consistent with the human studies [42]. Below we will discuss how many codons are affected by these bases substitutions by causing nonsense or missense mutations.

**Table 3:** Exonic base substitution types of *APC* and 10 other genes in canine colorectal tumors.*

Substitution type	Adenoma				Adenocarcinoma			
	APC		Other 10 genes		APC		Other 10 genes	
	Total	%	Total	%	Total	%	Total	%
C:G→T:A	56	22.49	181	24.46	103	18.69	349	25.16
C:G→G:C	25	10.04	141	19.05	87	15.79	239	17.23
C:G→A:T	38	15.26	114	15.41	71	12.89	218	15.72
T:A→C:G	50	20.08	113	15.27	95	17.24	209	15.07
T:A→G:C	34	13.65	106	14.32	96	17.42	217	15.65
T:A→A:T	46	18.47	85	11.49	99	17.97	155	11.18

* See Files S1 & S2 for detailed information for each gene.

#### Nonsense mutations

For *APC*, a total of 24 base substitutions (11 for adenomas and 13 for adenocarcinomas) caused nonsense (stop codons) mutations for 5 codons in 2 adenomas and 10 codons in 8 adenocarcinomas (Tables 1 and 2). For the non-*APC* 10 genes, a total of 81 base substations (22 for adenomas and 59 for adenocarcinomas) resulted in 17 nonsense mutations for 6 genes in 6 adenomas and 51 nonsense mutations for 7 genes in 15 adenocarcinomas. Hence, nonsense mutations were slightly more frequent in these non-*APC* genes in adenocarcinomas (Tables 1 and 2).

#### Missense mutation

We found that 708 substitutions (216 for adenomas and 492 for adenocarcinomas) resulted missense mutations for 133 codons in 8 adenomas and 338 codons in 15 adenocarcinomas, some of which will undoubtedly be germline and not cancer-related. For the other 10 genes, a total of 1,742 substitutions (612 for adenomas and 1,130 for adenocarcinomas) resulted in missense mutations for 445 codons in 10 genes and 8 adenomas and 834 codons in 10 genes and 15 adenocarcinomas, with a frequency approximately the same as that of *APC* (Tables 1 and 2).

## Discussion

*APC* is the best-known human colorectal tumor suppressor gene, with *APC* inactivation occurring in the vast majority of human colorectal tumors and being the earliest event yet identified in human sporadic colorectal tumorigenesis [16]–[33], [42]. Similar to their human counterparts, we report herein that *APC* is also frequently altered in sporadic canine colorectal tumors and that its alteration also appears to be an early event in canine colorectal tumorigenesis. Specifically, we recurrently detected large deletions, small indels, and an overall significantly higher mutation rate in both adenomas and adenocarcinomas in *APC* coding exons, in comparison with 10 other genes known to be altered in human CRC or other human cancers.

The study also revealed other homologies in *APC* aberrations between human and dog colorectal tumors. First, large deletions of ≥10-bases long were very frequent in canine *APC* coding exons in both adenomas and adenocarcinomas, at a rate of >18 times higher than the other 10 genes (Tables 1 and 2). Single-base or two-base deletions were also more common in canine *APC*, albeit not as outstanding as large deletions. The base substitution rate, on the other hand, was approximately the same between *APC* and other genes examined. These observations are consistent with many studies reporting recurrent allelic loss or loss of heterozygosity (LOH) of *APC* in human colorectal tumors [16]–[33], [42]. Second, in canine tumors, a substantial portion of the large deletions clustered at codons 1143–1250, spanning the 15 aa repeats region and downstream (Fig. 2). Similar to alterations within the human *APC* MCR [30]–[32], [42], these large deletions will mostly likely disrupt the β-catenin-binding sites in the 15 aa repeats region as well as in the 20 aa repeats region downstream (Fig. 2). Indeed, altered β-catenin protein expression has been noted in canine colorectal tumors [35], [43]. Third, a significant fraction of the small deletions was found within the armadillo domain of *APC* in canine tumors (Fig. 2), consistent with a mouse model study indicating that the armadillo domain can suppress intestinal tumorigenesis [44]. Lastly, a lower frequency of G:C→A:T mutations was observed for canine *APC*, similar to its human counterpart [42], although a cleaner comparison for base substitution would require the exclusion of germline mutations.

With only 23 canine colorectal tumors (8 adenomas and 15 adenocarcinomas) being sequenced and another 12 for qPCR validation, we acknowledge that our sample size is small. However, because of the high frequency of *APC* alterations, particularly deletions, as shown in Tables 1 and 2, we believe that this sample size is large enough to reach our conclusions discussed above.

This work, along with our previous genome-wide copy number abnormality study [15], provides critical evidence supporting that sporadic canine tumors are likely to share similar molecular pathogenesis pathways as their human counterparts. Hence, these studies lay the crucial molecular foundation justifying the use of sporadic canine CRCs in basic and clinical research to understand and treat human CRCs.

## Supporting Information

File S1 Detailed mutation statistics for each of *APC* and the other 10 canine genes from exon-resequencing analysis with the canine adenomas listed in Table S1.
(TXT)

File S2 Detailed mutation statistics for each of *APC* and the other 10 canine genes from exon-resequencing analysis with the canine adenocarcinomas listed in Table S2.
(TXT)

File S3 Detailed information for each mutated codon of *APC* and the other 10 canine genes in the adenomas listed in Table S1, including large deletions, small indels, nonsense mutations, missense mutations, and base substitutions.
(TGZ)

File S4 Detailed information for each mutated codon of *APC* and the other 10 canine genes in the adenocarcinomas listed in Table S2, including large deletions, small indels, nonsense mutations, missense mutations, and base substitutions.
(TGZ)

Table S1 Canine colorectal adenoma and adenocarcinoma samples used for bi-directional exon-resequencing analysis.
(XLS)

Table S2 Canine colorectal tumor and normal tissue samples used for qPCR analysis.
(XLS)
